# Bio-Accessibility of Phenolic Compounds from Green Banana-Fortified Bread During Simulated Digestion and Colonic Fermentation

**DOI:** 10.3390/molecules30183743

**Published:** 2025-09-15

**Authors:** Yasmeen M. Bashmil, Frank R. Dunshea, Rudi Appels, Hafiz A. R. Suleria

**Affiliations:** 1 Faculty of Human Sciences and Design, Department of Food and Nutrition, King Abdulaziz University, Jeddah 21589, Saudi Arabia; ybashmil@student.unimelb.edu.au; 2 Faculty of Science, School of Agriculture, Food and Ecosystem Sciences, The University of Melbourne, Parkville, VIC 3010, Australia; fdunshea@unimelb.edu.au (F.R.D.); rudi.appels@unimelb.edu.au (R.A.); 3 Faculty of Biological Sciences, University of Leeds, Leeds LS2 9JT, UK

**Keywords:** bio-accessibility, digestibility, functional bread, fermentation, green banana, HPLC-PDA, polyphenols, SCFAs

## Abstract

Functional foods are gaining heightened popularity in diet modifications. Green bananas contain a significant quantity of resistant starch, dietary fibre, and phytochemicals that demonstrate strong antioxidant properties, particularly due to the high concentration of polyphenols. The community demand for incorporating these essential components into food products, such as bread, has increased. Therefore, the aim of this study was to evaluate the differences in the content and bio-accessibility of phenolic compounds in bread enriched with 5, 10, and 15% of Australian green banana powder (GBF) from (Cavendish “*Musa acuminata*”, Ladyfinger “*Musa paradisiaca* L.”, and Ducasse “*Musa balbisiana*”), as well as their antioxidant capabilities and the generation of short-chain fatty acids (SCFAs) after in vitro gastrointestinal digestion and colonic fermentation. The 15% Cavendish bread exhibited significant TPC and TFC at 1.31 mg GAE/g and 0.05 mg QE/g, respectively, along with substantial antioxidant activity (DPPH, 0.40 mg TE/g), observed following stomach and intestinal phases. However, the 15% Ladyfinger bread exhibited the highest TTC following the stomach digestion, with 17.4 mg CE/g. The bio-accessibility of most phenolic components from 10% GBF-bread was elevated following the gastric and intestinal phases. Nonetheless, a substantial total phenolic content (50.3% in Ladyfinger bread) was still observable in the residue during colonic fermentation. The highest SCFAs production occurred in Cavendish and Ducasse bread after 24 h of fermentation. Overall, the consumption of GBF bread can positively influence intestinal health and provide antioxidant properties, facilitating future advancements in the creation of nutrient-dense and health-enhancing bakery products.

## 1. Introduction

Bread is a fundamental food intimately connected to our daily life. Although a variety of bread types are available, white bread remains the preferred option among consumers due to its sensory attributes, despite being classified as a high-glycaemic-index item. This classification arises from its high content of quickly digested starch. In addition, the removal of bran during the manufacture of white wheat flour leads to considerable loss of fibre, vitamins (notably vitamin E and B vitamins), minerals (including magnesium and iron), and antioxidants, in contrast to whole wheat flour [1]. Consequently, there is growing interest in fortifying bread with diverse dietary fibres (DF) and functional substances to leverage its efficacy as a carrier for health-enhancing elements. Recently, bran, vitamins, prebiotics, minerals, and other functional components have been proposed as partial substitutes for wheat flour [2,3,4,5]. Despite such progress, limited attention has been given to tropical fruit-based flours such as green banana flour (GBF), which combine sustainability benefits with bioactive potential.

Banana is a tropical fruit that includes various species of the genus *Musa* within the family *Musaceae*. It is currently ranked among the most cultivated fruit crops in the world, with global production reaching approximately 135 million tonnes in 2022 [6]. Unripe bananas exhibit significantly higher levels of flavonoids, DF, and resistant starch (RS) than ripe bananas [7]. Additionally, they demonstrate a superior antioxidant potential compared to other grains, vegetables, and herbs, ascribed to the abundance of phenolics, flavonoids, carotenes and other phytochemicals [8]. The flour derived from whole green bananas has also been proposed to increase the value of banana harvests by reducing waste generated throughout the production process, promoting sustainability, and retaining valuable nutrients lost during maturation [9]. The health benefits and biological effects of phenolic compounds are thoroughly established. Nonetheless, the bio-accessibility and consistency of these substances through digestion and absorption significantly influence their health benefits. This makes GBF a promising functional ingredient not only for improving nutritional value but also for addressing global food waste challenges.

Bioactivity and bio-accessibility are essential variables for the bioavailability of bioactive compounds, commonly used to assess the major nutritional effectiveness of living beings [10]. The nutrients and bioactive compounds’ bio-accessibility during and following food ingestion can be evaluated by simulating physiological conditions through simulated in vitro digestion [11]. Recent research has evaluated the effect of in vitro digestion on the levels of phenolic compounds and antioxidant capacity in different fruits and vegetables [12]. However, there is a paucity of research conducted on the simulated in vitro digestion and bowel fermentation of green banana-enriched bread. Many previous investigations into the stability within the gastrointestinal (GI) tract and the bioavailability of dietary phenolics post-GI digestion have shown significant variability among different polyphenols, depending on the quantity released from the dietary matrix [13,14].

Interestingly, about 90–95% of ingested polyphenols remain unabsorbed in the upper GI tract and reach the colon intact [15]. As phenolic compounds, they can engage in various interactions, such as ionic and hydrogen bonds, and hydrophobic interactions, with food matrix components [16]. In the colon, they can regulate the composition and function of local microorganisms and membrane permeability [17], hence minimize the proliferation of pathogenic species in the gut [18]. Furthermore, polyphenols can be metabolized by the gut microbiome into bioactive, low-molecular-weight constituents, particularly short-chain fatty acids (SCFAs), which are easily absorbed and provide many physiological benefits. Their potential as nutraceutical components has been verified by numerous clinical studies, which have confirmed their health-enhancing effects as antioxidants, antibacterial, anti-inflammatory and neuroprotective compounds [19].

To the best of our knowledge, no studies have systematically evaluated how simulated GI digestion and colonic fermentation affect phenolic bio-accessibility and SCFAs production in GBF–fortified bread. Addressing this gap is significant, as understanding these processes is critical to demonstrate the functional food potential of such products, and provides preliminary evidence of the nutritional and functional value of GBF. Therefore, the aim of this research was to evaluate the antioxidant capacity and bio-accessibility of various phenolic compounds from GBF-bread produced by substituting wheat with unripe Australian-grown Cavendish, Ladyfinger, and Ducasse banana flour, utilizing a static in vitro method for GI digestion and gut fermentation.

## 2. Results and Discussion

### 2.1. Changes in Phenolic Compounds and Bioactivity During In Vitro Digestion

#### 2.1.1. Impact of In Vitro Digestion on Available Total Phenolic, Flavonoid, and Tannin Content

**Figure 1:**
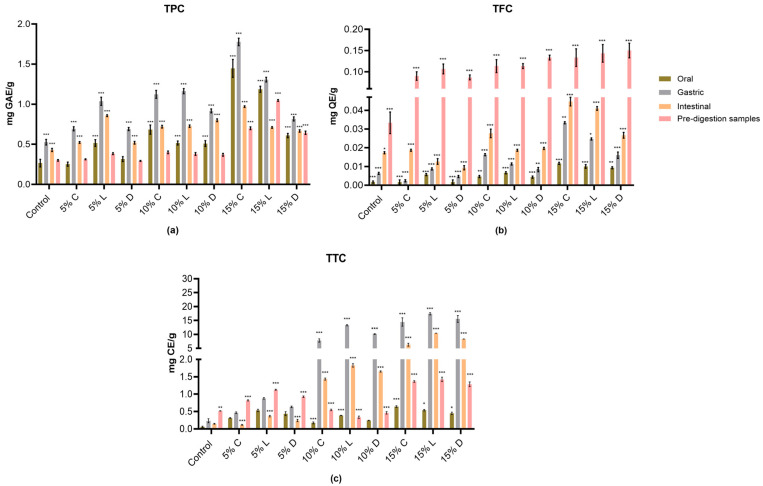
The evaluation of phenolic content in the in vitro digestates of wheat-GBF substitutes at 5%, 10%, and 15% concentrations. All assays’ findings were reported as mean ± standard deviation (n = 3) on a dry weight basis, with control values deducted. (**a**): total phenolic content (TPC); (**b**): total flavonoid content (TFC); (**c**): total tannins (TTC); C: Cavendish; L: Ladyfinger; D: Ducasse; pink bars: pre-digestion samples; green bars: oral phase; grey bars: gastric phase; yellow bars: intestinal phase; GAE: gallic acid equivalents; QE: quercetin equivalents; CE: catechin equivalents; *: (*p* ≤ 0.05); **: (*p* ≤ 0.01); ***: (*p* ≤ 0.001).

Figure 1 presents the analysis of total phenolic content (TPC), total flavonoid content (TFC), and total hydrolysable tannin content (TTC) of the control and banana bread samples enriched with varying amounts (5%, 10%, and 15%) of GBF (Cavendish, Ladyfinger, and Ducasse cultivars) during in vitro digestion stages (oral, gastric, and intestinal). The pre-digestion samples were also analyzed for comparison. The results demonstrated that the available TPC and TTC of all banana bread samples significantly increased during in vitro digestion. In contrast, the TFC exhibited a notable decrease compared to the pre-digestion samples. The phenolic concentration was variably affected by each stage of digestion, especially in bread with the 15% banana substitute (Figure 1).

The TPC analysis showed significant increases with the addition of more banana flour up to 15% across all banana cultivars after oral, gastric and intestinal digestion compared to the control bread and the pre-digestion samples (*p* ≤ 0.001) (Figure 1a). The gastric phase exhibited the most significant release of phenolics for all samples, particularly, in bread samples containing 15% Cavendish and Ladyfinger flour (1.78 and 1.31 mg GAE/g, respectively). This highlights the critical role of the stomach’s acidic environment in liberating these compounds and the high stability of phenolic compounds under low pH conditions [20]. Although the phenolics released during intestinal digestion were significantly lower than during the gastric phase, the intestinal stage retained a substantial proportion of TPC compared to the pre-digestion samples with the greatest TPC in the 15% Cavendish bread (0.97 GAE/g).

The statistical analysis demonstrated that TPC was strongly influenced by both the level of banana flour substitution and the digestion phase (*p* < 0.001). A significant interaction between cultivar and substitution level (*p* < 0.001) further indicated that the effect of enrichment depended on the variety used. Among the cultivars, 15% Cavendish consistently yielded the greatest TPC values across digestion stages, whereas Ladyfinger and Ducasse produced smaller but still significant enhancements.

Intestinal enzymes may act on food residues to enhance the release of phenolic compounds and boost their overall quantity [21,22]. Additionally, another study indicated that around 30 to 48% of chlorogenic acids may be digested in the small intestine. The residual fragments may be further broken down in the colon. The associated hydroxycinnamates, including ferulic, caffeic, and quinic acids, may result from the breakdown of chlorogenic acids, hence enhancing the TPC value [23]. The enhancement of the TPC may occur because, during intestinal digestion, the hydrophobic interactions between carbohydrates and phenolic compounds can be reduced in the acidic pH below 6.9, alongside the action of α-amylase, lipase, pancreatin, and bile salts, which together enhance phenolic compound bio-accessibility and transport [24]. Moreover, the pH of the small intestine gradually shifts from ~6.7 in the proximal region to ~7.5 in the terminal ileum, and this shift, together with longer transit time, could increase microbial density. While microorganisms were not included in our in vitro digestion model, in vivo microbial activity is of considerable significance, as it may further influence the release and transformation of phenolic compounds [25]. In this study, the increase in TPC probably results from enzyme activity modifying the dietary matrix, so facilitating the release of greater quantities of phenolic compounds. As a result, post-digestion assessments revealed a greater bio-accessible phenolic content, potentially improving biological functions.

No research has investigated the impact of in vitro digestion on the phenolic content in wheat-GBF bread. However, compared to other food products, the research by Pico et al. [26] showed that cakes manufactured from wheat and banana flour exhibited a significantly higher TPC after simulated digestion than before digestion. Likewise, the present data were consistent with the results of Koehnlein et al. [27], who performed a comparative analysis of phenolic compounds before and after simulated food digestion across diverse food categories, including corn starch, rice flour, cereals, tapioca starch, pasta, nuts, whole wheat bread, fruit, vegetables, and chocolate. Their research indicated a significant increase in phenolic content post-digestion, with cereals exhibiting a fivefold increase and legumes increasing by threefold. The rise in phenolic compounds is attributed to enzymes involved in the digestion of carbohydrates and proteins. Notably, grains and flour exhibited a greater release of phenolic compounds following digestion compared to other foods.

On the other hand, the results for TFC demonstrated significant changes in flavonoid bio-accessibility across different digestion phases as shown in Figure 1b. The pre-digestion samples exhibited the highest TFC values across all bread formulations (*p* ≤ 0.001). Following in vitro digestion, the intestinal phase showed the highest flavonoid release among the digestion stages, particularly in the 15% Cavendish addition (0.05 mg QE/g), followed by the 15% Ladyfinger (0.04 mg QE/g). The gastric phase also contributed to flavonoid liberation, but to a lesser extent compared to the intestinal phase. In contrast, the oral phase had the lowest TFC values, indicating minimal flavonoid release at this stage. Among the enriched bread samples, 15% Cavendish flour substitution, exhibited the highest flavonoid retention throughout digestion. In addition, the two-way ANOVA showed that both the level of GBF substitution and the banana cultivar significantly influenced TFC, with a strong interaction effect between the two factors (*p* < 0.001). This indicates that the flavonoid content was not determined by substitution level or cultivar alone, but rather by their combined effect, where higher substitution levels (particularly 15% Cavendish and Ladyfinger) consistently enhanced flavonoid release compared with lower levels across all cultivars.

These findings highlight the positive impact of GBF on flavonoid stability and bio-accessibility, suggesting its potential role in improving the antioxidant properties of functional bread products. Compared to our previous work, TFC in Cavendish, Ladyfinger, and Ducasse followed a similar trend, with the highest flavonoid release occurring during the intestinal phase. The consistent pattern across banana cultivars highlighted the importance of digestion in liberating bound flavonoids, increasing their potential bioavailability [28].

This was consistent with the findings of Chait et al. [29], who investigated the effect of simulated GI digestion on carob polyphenols. They noted a minor alteration in carob polyphenols during the oral phase compared to other digestion stages, likely due to the brief contact time (2 min), the restricted solubility of these compounds in saliva, and the relatively low α-amylase activity (1.25 µkat/mL), which initiates starch hydrolysis in the mouth.

In contrast to TPC, the intestinal TFC was markedly elevated compared to the oral and stomach phases, possibly due to the improved liberation of flavonoids from the food matrix during intestinal digestion. Our findings aligned with those of Wu et al. [30], whose study revealed a dramatic rise in the TFC of both light and dark-roasted coffee beans following the intestinal digestive phase. As stated by Ortega et al. [31], physicochemical alterations, including oxidation or interactions with other molecules, such as polysaccharides in the digestive mixture, could explain the reduction in phenolic compounds during in vitro digestion. Moreover, a decrease in phenolic compounds may occur due to the precipitation of specific phenolics, especially tannins, through binding with enzymes during digestion. Supporting this, González-Sarrías et al. [32] indicated that both protein and pH conditions limited the bioavailability of ellagic acid and ellagitannins.

The analysis of TTC revealed notable increase in tannin bio-accessibility in banana enriched bread after GI digestion compared to the pre-digestion samples and control bread (*p* ≤ 0.001) (Figure 1c). The gastric phase displayed the highest tannin release with 17.4 mg CE/g in the 15% Ladyfinger bread. However, a considerable amount of tannins remained available during the intestinal phase, suggesting their potential for absorption, while the oral phase exhibited the lowest TTC, indicating minimal tannin release at this stage. Bread enriched with 10% and 15% GBF, particularly from Ladyfinger, showed the greatest tannin retention throughout digestion.

The two-way ANOVA further confirmed that both the level of banana flour substitution and the digestion phase significantly influenced TTC (*p* < 0.001). Importantly, there was also a significant interaction effect between flour level and banana cultivar (*p* < 0.001), indicating that the extent of tannin release depended not only on the substitution percentage but also on the cultivar used. This interaction highlights that varieties such as Ladyfinger responded more strongly to higher substitution levels, particularly in the gastric phase, compared to Cavendish and Ducasse, thereby emphasizing cultivar-specific effects on tannin bio-accessibility.

A study by Fabbrini et al. [33] found that acidic circumstances can significantly contribute to the degradation of the food matrix and promote tannin solubilization. Specifically, hydrolysable tannins can be decomposed under mildly acidic circumstances, releasing monomers including ellagic or gallic acid in the upper digestive system, which are eventually absorbed. This process highlights the importance of the stomach’s acidic environment in facilitating the release and potential absorption of tannins.

Corresponding to our results, Quatrin et al. [34] found that soluble tannins in Jaboticaba fruits exhibited the most significant reduction following salivary digestion, exceeding 74%. Furthermore, around 80–90% of these hydrolysed tannins were decomposed following stomach and intestinal digestion. Additionally, Spencer et al. [35] reported that procyanidin oligomers (trimer to hexamer) may breakdown and hydrolyse into epicatechin monomers and dimers in the acidic environment of the stomach. Nonetheless, Hollman [36] claimed that only proanthocyanidins consisting of fewer than three catechins were likely to be absorbed by the intestine. On the contrary, a slightly alkaline environment could improve the hydrophobic interactions between proteins and tannins, resulting in precipitation [22].

#### 2.1.2. Evaluation of Antioxidant Capacity

**Figure 2:**
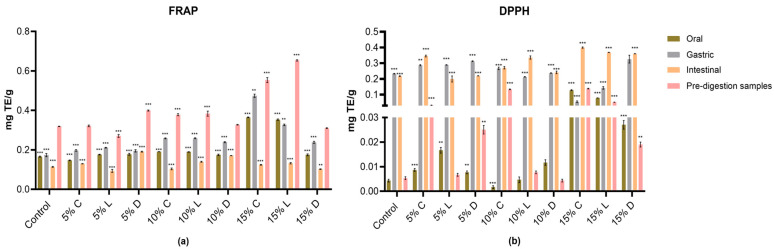
The evaluation of antioxidant capacity of wheat-GBF substitutes (5%, 10%, and 15%) in vitro digestates. The results of all assays were expressed as mean ± standard deviation (n = 3) on a dry weight basis, with the control values subtracted. (**a**): ferric reducing antioxidant power assay (FRAP); (**b**): 2,2-diphenyl-1-picrylhydrazyl antioxidant assay (DPPH); C: Cavendish; L: Ladyfinger; D: Ducasse; pink bars: pre-digestion samples; green bars: oral phase; grey bars: gastric phase; yellow bars: intestinal phase. TE: Trolox equivalents; **: (*p* ≤ 0.01). ***: (*p* ≤ 0.001).

In this study, Antioxidant activity across different in vitro digestion phases was assessed using both the DPPH and FRAP tests. The DPPH method is based on an electron transfer reaction that measures the scavenging ability against nitrogen free radicals. In contrast, the FRAP assay does not involve free radical quenching but instead quantifies the reduction in ferric ions (Fe^3+^) to ferrous ions (Fe^2+^) [37]. This complementary approach enabled a broader assessment of antioxidant mechanisms. As shown in Figure 2, banana–wheat bread samples subjected to in vitro digestion exhibited distinct antioxidant responses, with the 15% substitution level demonstrating the greatest overall activity, particularly in terms of free radical scavenging and ferric ions reduction.

The FRAP assay results revealed marked differences in antioxidant activity among the various banana bread formulations and throughout the in vitro digestion phases (*p* ≤ 0.001), as shown in Figure 2a. Pre-digestion samples showed the highest antioxidant potential with formulations containing 15% banana flour, especially from Ladyfinger flour (0.65 mg TE/g), compared to other cultivars. During in vitro digestion, the gastric phase produced the most substantial release of antioxidants, with the greatest FRAP in the 15% Cavendish bread (0.47 mg TE/g). Although the intestinal phase maintained considerable antioxidant activity, it exhibited a slight decline relative to the gastric phase, likely due to structural modifications or degradation of the antioxidant molecules [38]. In contrast, the oral phase displayed the lowest antioxidant capacity, which suggest minimal antioxidant release at this initial stage. A significant interaction effect (*p* < 0.001) was observed in FRAP results, showing that the antioxidant response to banana flour enrichment differed depending on the cultivar and level used. This interaction highlights that while Ladyfinger flour maximized pre-digestion FRAP values, Cavendish flour responded more strongly during gastric digestion, reflecting cultivar-specific antioxidant behaviour. Overall, banana-enriched bread formulations demonstrated significantly higher FRAP compared to the control bread.

On the other hand, the DPPH radical scavenging activity results demonstrated significant increase after gastric and intestinal phases compared to pre-digestion samples (Figure 2b). The pre-digestion samples exhibited lower antioxidant activity compared to the digested samples, which indicate that the digestion process could enhance the release of antioxidant compounds. Among the digestion stages, the intestinal phase exhibited the highest DPPH scavenging activity across all bread samples, with the 15% Cavendish-enriched bread had the greatest DPPH activity at 0.40 mg TE/g (*p* ≤ 0.001). However, the gastric phase showed less antioxidant activity than the intestinal phase, it also contributed significantly to antioxidant release, whereas the oral phase displayed the lowest antioxidant capacity. Bread samples enriched with 15% Ducasse bread showed the highest antioxidant activity during gastric digestion (0.33 mg TE/g) when compared to Cavendish and Ladyfinger bread. These discrepancies may be attributable to variances in banana cultivars.

Statistical analysis confirmed that all three factors, digestion phase, level of flour substitution, and banana cultivar, had significant effects on DPPH activity (*p* < 0.001). In addition, a strong interaction was identified (*p* < 0.001), indicating that the antioxidant potential was shaped by the combined influence of cultivar, substitution level, and digestion stage. This pattern emphasizes that while the 15% Cavendish promoted maximum antioxidant activity in the intestinal phase, the 15% Ducasse was more effective during gastric digestion, which suggests that the functional benefits of GBF are cultivar dependent.

Results from our current study on raw wheat and GBF bread align with the recent research conducted to evaluate the impact of incorporating 10% GBF on the prebiotic potential, antioxidant capability of noodles [39]. The GBF fortified noodles revealed a significant (*p* < 0.05) increase in antioxidant capacity, as indicated by elevated DPPH and FRAP activities, compared to the control noodles [39]. In another investigation, large-leaf yellow tea bread was created from ultrafine large-leaf yellow tea powder and standard wheat flour as the primary ingredients. At the 3% replacement level, the antioxidant capacity (DPPH) of large-leaf yellow tea bread was significantly greater compared to that of the control bread [40]. Furthermore, Gawlik-Dziki et al. [41] examined the impact of adding onion skin powder on the antioxidant capabilities of bread. Their findings indicated that the antiradical activity, chelating capacity, and lipid peroxidation inhibition of bread containing 5% onion skin powder were substantially greater than those observed in the control during GI digestion.

Various factors can affect the antioxidant activity and the release of phenolic compounds during GI digestion. Green bananas are rich in RS and DF, which can encapsulate antioxidant compounds, thereby affecting and delaying their release during digestion. The structural integrity of these components may hinder the bio-accessibility of antioxidants, as they are trapped within the food matrix [28]. Furthermore, the drying method can strongly impact the availability of polyphenols and the antioxidant activity in bread samples. As found by Sarkar et al. [42], in comparison to other drying methods, for example hot air drying, sun drying, and microwave drying, freeze-drying maintains the TPC to the greatest extent. Freeze-dried mango skins had a TPC of 7.2 ± 0.06 mg/g dry weight, while sun-dried mango skin samples showed a significantly lower TPC of 4.1 ± 0.03 mg/g dry weight.

Moreover, the interactions between polyphenols, gluten, RS, and DF during bread-making can significantly influence the bioavailability and digestion of polyphenols. Polyphenols can bind to gluten proteins, potentially reducing their bioavailability. This binding could prevent the release and absorption of polyphenols throughout digestion. Flavonoids, tannins, and coumarins have displayed a tendency to form insoluble complexes with gluten proteins [43]. Further, polyphenols can form complexes with DF and RS, which may reduce their bio-accessibility. These interactions may result in the creation of colloidal structures and chemical complexes, potentially decreasing the absorption of polyphenols in the intestine. While these interactions can reduce the immediate bioavailability of polyphenols, they may also facilitate the delivery of these compounds to the colon, where they can exert prebiotic effects and undergo microbial metabolism, contributing to gut health [44].

Dough fermentation during bread preparation significantly influences the antioxidant activity and phenolic content of the bread. The metabolic activities of fermenting microorganisms, such as yeasts and lactic acid bacteria, can modify the phenolic profile and enhance the antioxidant capabilities of the final product. Arjmand et al. [45] demonstrated that fermentation can increase the TPC in dough. Their research on quinoa dough fermented with *Lactiplantibacillus plantarum* subsp. *Plantarum* observed a 170% increase in TPC after 12 h of fermentation. This enhancement was attributed to microbial enzymatic activities that release bound phenolic compounds from the food matrix, increasing their bio-accessibility. The increase in phenolic compounds during fermentation correlates with enhanced antioxidant activity. In the same study, the fermented quinoa dough exhibited a 105% increase in FRAP after 12 h. This suggests that the liberated phenolics possess significant antioxidant potential, contributing to the overall health benefits of the bread.

The duration of fermentation is essential in determining the degree of these improvements. Prolonged fermentation has been associated with increased TPC and antioxidant activity. For example, doughs enriched with grape and pomegranate seeds showed higher TPC and Trolox Equivalent Antioxidant Capacity (TEAC) values with extended fermentation periods. This indicates that allowing sufficient fermentation time can maximize the release of phenolic compounds and boost antioxidant properties [46].

#### 2.1.3. Quantification of Phenolic Compounds in Wheat-Banana Bread Using High-Performance Liquid Chromatography Photodiode Array (HPLC-PDA)

HPLC-PDA is a commonly employed analytical method for quantifying phenolic compounds in food matrices. In this project, HPLC-PDA was employed to evaluate the phenolic profile of the enriched wheat bread with different levels (5%, 10%, and 15%) of unripe Cavendish, Ladyfinger, and Ducasse flour, compared to a control bread without substitution (Table 1). This analysis focused on key phenolic compounds, including gallic acid, catechin, chlorogenic acid, kaempferol, and quercetin, among others, to assess the effect of banana flour enrichment on TPC. The results provide insights into how different banana cultivars and substitution levels influence the retention and distribution of polyphenols in the final bread product. Addressing these variances is crucial for improving the functional and nutritional attributes of banana-enriched bread.

Minor variations observed in the levels of some phenolic compounds are partly attributable to natural variability in the raw materials. In particular, genetic differences among banana cultivars are known to influence their chemical composition, including phenolic content, as demonstrated in our previous studies [28,47]. These variations may also reflect the effect of different substitution levels (5–15%) of banana flour, which alter the relative proportion of banana-derived solids in the bread matrix. Such changes are not always linear due to processing-induced matrix interactions (e.g., polyphenol–protein/fibre binding and release) and thermal transformations during baking, which can lead to partial degradation, isomerisation, or hydrolysis of certain phenolics. Analytical sensitivity can further contribute to these small fluctuations, which are common in complex food systems [48]. Importantly, these minor differences do not affect the overall trends or interpretation of our results.

As shown in Table 1, the incorporation of GBF notably increased the concentrations of multiple phenolic compounds compared to the control bread, which exhibited the lowest values across all compounds (*p* < 0.05). Among the most abundant phenolics, gallic acid showed a significant increase, particularly in Ladyfinger (456 µg/g) and Ducasse (239 µg/g) bread at 15% substitution. This suggests that these banana cultivars are rich sources of this potent antioxidant. Similarly, protocatechuic acid was significantly higher in 15% Cavendish bread (2462 µg/g), indicating a strong cultivar-dependent variation in phenolic composition. However, chlorogenic acid, a major hydroxycinnamic acid [49], significantly increased in 15% Ladyfinger bread (645 µg/g) and 15% Cavendish bread (252 µg/g), which indicates that both cultivars could contribute to the retention of this compound during the bread-making process. Caffeic acid, another important hydroxycinnamic acid, showed the highest concentration in 15% Cavendish bread (39.2 µg/g). Similarly, coumaric acid was most abundant in 15% Cavendish bread (246 µg/g).

Furthermore, syringic acid has been linked to potential anti-diabetic and neuroprotective functions [50]. In this study, syringic acid was reported in the greatest quantity in the 15% Cavendish bread (62.5 µg/g), followed by Ladyfinger and Ducasse. Additionally, *p*-hydroxybenzoic acid is a naturally existing phenolic compound discovered in several plant-based foods, such as bananas, and plays an essential role in antioxidant activity and antimicrobial properties [47]. In banana-enriched bread, the presence of *p*-hydroxybenzoic acid varies depending on the substitution level and banana cultivar used. The HPLC analysis of banana bread formulations revealed that higher substitution levels (10% and 15%) generally resulted in increased concentrations of *p*-hydroxybenzoic acid, particularly the 15% Cavendish samples with 1536 µg/g. Moreover, *p*-hydroxybenzoic acid is linked to anti-inflammatory and antimicrobial activities, that could contribute to the extended shelf life and potential health benefits of banana bread [51]. However, the heat stability of *p*-hydroxybenzoic acid during baking may influence its final concentration, with some degradation possible due to thermal processing [52]. Despite this, banana flour incorporation effectively enhances the phenolic profile of bread, reinforcing its value as a functional food ingredient.

Studying the flavonoid content in wheat-banana bread, catechin, a well-known flavonoid with strong antioxidant properties [53], exhibited the highest concentration in Cavendish bread at 15% substitution (2148 µg/g), followed by Ladyfinger (379 µg/g) and Ducasse (327 µg/g) (Table 1). This trend suggests that the catechin content varies depending on the banana cultivar, with Cavendish being particularly rich in this compound. Additionally, quercetin and kaempferol, both flavonoids linked to anti-inflammatory and cardiovascular benefits [54,55], were significantly higher in 15% Cavendish bread, with quercetin reaching 71.0 µg/g and kaempferol at 91.6 µg/g, compared to the control, which had significantly lower values (17.5 µg/g for quercetin and 10.5 µg/g for kaempferol). Likewise, epicatechin, another strong antioxidant, showed substantial increases with higher banana flour substitution, with 15% Ducasse-enriched bread containing the highest levels (265 µg/g) (Table 1).

Comparably, diosmin had the highest level in the 15% Cavendish bread (15.5 µg/g). Furthermore, the stilbenoid compound [56], polydatin, exhibited notable increases with banana flour incorporation, reaching its highest level in 15% Cavendish bread (6.6 µg/g), as well as resveratrol which increased across all banana-enriched samples, with 15% Cavendish enriched bread showing the highest level (32.6 µg/g) compared to other banana cultivars and the control bread.

The high-water absorption capacity (WAC) of GBF, related to its starch granule structure, fibre, RS, can significantly influence the retention of phenolic acids and flavonoids in the GBF-enriched bread. This enhanced water retention in the dough matrix may protect these bioactive compounds from thermal degradation during baking, leading to higher retention in the final product. The GBF’s high WAC can stabilize phenolic acids during baking [5,57,58]. Research has shown that certain cooking methods that involve water, such as boiling and steaming, can preserve or even enhance the phenolic content in food products. For instance, a study on edible leaves demonstrated that boiling and steaming led to higher levels of polyphenols compared to frying, which caused a reduction in these compounds. This suggests that the presence of water during thermal processing can help in retaining phenolic acids [59]. Moreover, the moisture retained in the dough may create a protective environment that reduces the degradation of flavonoids caused by heat. As found by Blanch and Ruiz del Castillo [60], studying the influence of baking temperature on the polyphenols content and antioxidant efficacy of black corn bread found that certain phenolic compounds, including flavonoids, were better preserved at specific moisture levels during baking. This indicates that maintaining adequate moisture content during baking can contribute to the stability of flavonoids.

#### 2.1.4. Bio-Accessibility of Selected Phenolic Compounds in GBF-Enriched Bread

In Table 2, the bio-accessibility of fifteen phenolic compounds is shown during different digestion and fermentation stages of the 10% GBF-enriched bread. The expression “bio-accessibility” describes the amount of bioactive compounds that are ingested and have the capacity to be absorbed by the epithelial layer of the GI tract [10]. After a thorough analysis, it was discovered that the stomach and intestinal phases exhibited the highest bio-accessibility for most phenolic compounds. Following the colonic fermentation, the bio-accessibility of TPC was demonstrated to be at its lowest level, which is consistent with the results observed by de Almeida et al. [61]. They noted a consistent decrease in phenolic bio-accessibility, which reached its lowest value after 24 h of microbial fermentation. The concept that phenolic acids, which are the major phenolic compounds found in fruits and vegetables, are usually absorbed in their aglycone state in the upper part of the GI tract is supported by the findings of Saura-Calixto et al. [62]. According to a prior study, the stomach and small intestine may serve as the main pathways for the active absorption of phenolic acids, such as caffeic and gallic acids [63]. Therefore, it is possible to infer that the majority of phenolic acids could be released from the 10% banana-enriched bread as a result of GI digestion, which agrees with the findings of our study.

Furthermore, our investigation disclosed differences in the bio-accessible location and bio-accessibility of the same phenolic compounds across various banana varieties used in the enriched bread. For instance, Ducasse exhibited a significantly greater release of caftaric acid (74.0%) than other cultivars during the gastric phase. Simultaneously, cafatric acid bio-accessibility was enhanced during the intestinal stage after the consumption of the Cavendish and Lady-finger varieties. Tarko and Duda-Chodak [64] also confirmed that the bio-accessibility discrepancies of phenols are primarily due to the interactions between them and the food matrix. Phenolic compounds may modify the extractability and sensitivity to microbial metabolism and digestive enzymes when they bind to the food matrix with a range of formulations [65]. In addition to DF structure, Rodríguez-Roque et al. [66] found that the mineral content of the fruit juice might influence the interactions with phenolic compounds and, as a result, restrict the release of phenolic compounds. Additionally, it has been proposed that the bio-accessibility of phenolic compounds may be improved by the inclusion of sugar by mechanisms such as reducing interactions between pepsin and tannins or increasing the solubility of tannin-pepsin complexes. These findings provide strong justification for the significant polyphenol release from banana-fortified bread during the gastric and intestinal phases in this research.

As illustrated in Table 2, Cavendish bread exhibited the greatest bio-accessibility of gallic and *p*-hydroxybenzoic acids during oral digestion (101 and 100%, respectively) followed by protocatechuic, syringic, coumaric, and chlorogenic acid. It was found by Alves et al. [67] that the free phenolic compounds in bread have been determined after the oral phase. These compounds include three hydroxycinnamic acid derivatives (ferulic, caffeic, and rosmarinic acids) and two hydroxybenzoic acid derivatives (gallic and 3,4-dihydroxybenzoic acids). Generally, the bio-accessibility of free phenolics upon oral digestion was 91%, suggesting that they were almost completely dissolved in saliva due to their high solubility in water.

Theoretically, it has been proposed that the body is unable to effectively absorb about 60% of the chlorogenic acid observed in bananas. Rather, it necessitates additional enzymatic breakdown by gut microbiota during colonic fermentation to release caffeic acid. This is suspected to be the result of the absence of esterase enzymes [68]. According to the research carried out by Olthof et al. [69], the bio-accessibility of chlorogenic acid from the GI tract, especially the small intestine, was restricted to a maximum of 33% of the consumed quantity. It is important to note that the phenolic compounds concentration in all banana breads presented a significant increase following GI digestion. This result was in line with the findings of Ordoñez-Díaz et al. [70] who discovered that the polyphenols percentage in mangoes significantly increased after in vitro stomach digestion. These polyphenols, which are covalently bound to cell wall polysaccharides in the food matrix or occur as hydrogen bonding complexes, are released as a result of acidic conditions and the presence of pepsin in the stomach [71]. In accordance with our findings, Lucas-González et al. [72] found a significant elevation in gallic acid concentration in persimmons fruits after gastric digestion.

Following the intestinal digestion, the average polyphenol content in GBF bread had a significant rise. The reported rise can be mainly attributed to a significant increase in the amount of specific phenolic compounds throughout the intestinal phase, such as protocatechuic acid (from 87.2% to 91.0%) in Ducasse bread, caftaric acid, from 63.7% to 101% for Cavendish bread, chlorogenic acid (from 69.8% to 88.8%) for Cavendish bread and (from 71.4% to 78.7%) for Ladyfinger bread, caffeic acid (from 54.7% to 81.7%) in Ducasse bread, and syringic acid which climbed from 92.0% to 96.2% in Cavendish bread. The breakdown of weak bonds between supramolecular structures within the dietary matrix and polyphenols may be affected by different factors at the intestinal level, including pH, pancreatin activity, and the availability of bile salts. This phenomenon is especially significant for polyphenols with low molecular weights, as it enhances the bioavailability of these polyphenols and facilitates their release from the food matrix during digestion [73]. It is possible that all these molecules are potentially absorbed after completing the intestinal digestion. In research performed by Jara-Palacios et al. [74], the phenolic composition of various white winemaking byproduct extracts (grape pomace and its components: skins, seeds and stems) was assessed following in vitro GI digestion. They discovered that in the intestinal digesta, the protocatechuic acid content of wine production leftovers was higher than in the pre-digestion samples. The breakdown of flavonoids may have been the reason of the high protocatechuic acid concentration during digestion [75].

Our study on the impact of GI digestion on the bio-accessibility of flavonoids demonstrated that the intestinal phase had various advantages over the gastric stage, leading to increased flavonoid levels. The possible interaction between protease and flavonoids at low pH levels may result in the production of flavonoid-protease complexes. In agreement with some previous research, the binding ability of digestive enzymes and catechins may be affected by the pH levels in the gastric or intestinal environment [76]. Acidic stomach conditions led to a decrease in the solubility of flavonoids due to the interaction of flavonoids with pepsin. Furthermore, trypsin facilitated the release of phenolic compounds by liberating protein-bound phenolics, and the reduction in the chyme particle size positively influenced the release of these compounds.

Another possible aspect may be the physical embedding caused by DF. The interaction between phenolic compounds and DF may occur because of the presence of hydrophobic aromatic rings and hydrophilic hydroxyl groups in phenolic compounds [30]. Phenolic compounds interact with polysaccharides primarily through hydrogen bonds, where the hydroxyl groups of the phenolics bind to the oxygen atoms present in the glycosidic linkages of complex carbohydrates. In addition, covalent bonds and hydrophobic interactions, including ester bonds, are also involved. The breakdown of these bonds requires a contribution of bacterial enzymes [77].

This study indicated that the primary flavonoid components in GBF bread were quercetin, catechin, and its isomer epicatechin. Catechin and epicatechin serve as the fundamental components of condensed tannins, which may be generated during tannin digestion. The findings indicated that these flavonoids increased significantly following intestinal digestion in the bread fortified with the three banana cultivars. Also, the precursor molecule for tannins, gallic acid, can be synthesized via the hydrolysis of chlorogenic acids and tannins. During all stages of digestion, gallic acid continually displays significant bio-accessibility [78].

#### 2.1.5. Estimation of Short Chain Fatty Acids (SCFAs)

**Figure 3:**
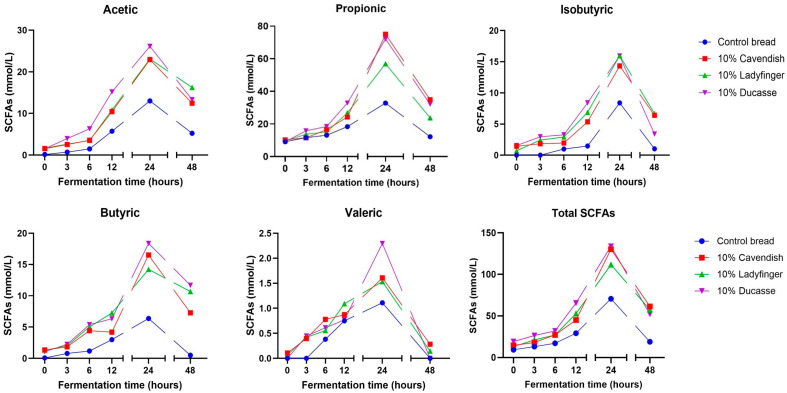
The pattern of SCFAs production in 10% GBF–wheat bread digesta after a complete colonic fermentation. The standard errors of the differences (SED) for the interaction between treatment and time are as follows: acetic acid: 0.3757; propionic acid: 0.6599; iso-butyric acid: 0.2894; butyric acid: 0.4949; and valeric acid: 0.08228; total SCFAs: 0.9486.

The concentration of five SCFAs (acetic, butyric, iso-butyric, propionic, and valeric acid) over time in the gut fermented wheat bread and 10% GBF enriched bread samples was shown in Figure 3. Primarily, propionic acid was the predominant SCFA after the fermentation of all banana bread samples, followed by acetic acid and iso-butyric acid. These results contradict a previous study by Shi et al. [79], which identified acetic acid as the most abundant SCFA after the simulated colonic fermentation of various lettuce varieties. The dissimilarity may be attributed to the presence of different types of DF. DF may affect the amount and variety of SCFAs produced in the colon [80]. Previous research by Welli et al. [81] supports our results, confirming that supplementing the diet with Cavendish banana flour resulted in increased propionic acid synthesis.

Subsequent to the GI digestion, GBF breads showed the capacity to retain substrates, including polyphenols, which are metabolizable by gut microbes. GBF–wheat bread contains substantial amounts of DF and RS in comparison to the control bread, as previously demonstrated in the study by Bashmil, Bekes, Ruderman, Suleria, Appels and Dunshea [57]. Carbohydrate fermentation is the primary source of energy for the gut microbiome, resulting in the formation of SCFAs. These acids are associated with decreased pH levels and gas production [82]. Correspondingly, recent findings from in vitro colonic fermentation of a tannin-rich extract confirm the hypothesis that tannins can operate as an additional substrate to produce SCFAs. In fact, earlier studies have shown that tannins have more significant effects than inulin on the generation of acetic, butyric, and propionic acids during the in vitro fermentation tests using human stool [83].

The fermented Cavendish bread demonstrated the highest propionic acid concentration (75.6 mmol/L), followed by Ducasse and Ladyfinger with 72.1 and 56.9 mmol/L, respectively. Significant differences in the synthesis of SCFAs were observed among the various banana breads. The generation of all SCFAs during the fermentation of 10% banana bread peaked at 24 h of simulated colonic fermentation. Nonetheless, Ducasse and Cavendish produced the highest concentration of the majority of the examined SCFAs. On the other hand, the Ladyfinger bread exhibited the highest generation of iso-butyric acid following 24 h of fermentation. The synthesis pattern of SCFAs in all banana bread samples was similar, particularly after 12 h of fermentation.

The microbial fermentation of DF primarily produces SCFAs, including acetate, propionate, and butyrate measured here. These compounds have a significant influence on energy metabolism, immune function, and intestinal cell proliferation in the body [84]. Also, SCFAs may maintain the optimal function of the large intestine and protect it from pathological circumstances by establishing an acidic environment in the colon, particularly highlighting butyric acid [85]. The production of SCFAs is mainly linked to the degradation of carbohydrates, especially RS and DF, as well as particular bacterial species in the colon [86,87]. According to Wong et al. [86], the generation of SCFAs is affected by the substrate source and the transit time within the colon. The indigestible DF in unripe bananas is more abundant in Cavendish and Ducasse compared to Ladyfinger, as previously reported in our earlier research [28]. As noted in Figure 3, our data indicated a substantial reduction in SCFAs levels in all banana-bread samples following 48 h of gut fermentation, which could be due to the depletion of DF, which serves as fuel for gut bacteria, or from the diminished microbiota diversity within the distal colon [88].

Nevertheless, it should also be noted that this study employed an in vitro digestion and colonic fermentation model as a preliminary approach to assess the bio-accessibility of phenolic compounds. While we acknowledge that in vivo conditions involve additional complexities such as absorption, metabolism, and host interactions that may alter compound stability, in vitro models are widely recognized as reliable pre-screening tools. Therefore, our findings should be interpreted as indicative, and future in vivo studies are required to validate these results under physiological conditions.

## 3. Materials and Methods

### 3.1. Production of Green Banana Flour

GBF was prepared following the procedure adapted from Bashmil et al. [28]. Whole, fresh, unripe bananas (Cavendish, Ladyfinger, and Ducasse cultivars) were sourced from local markets in Melbourne, Australia. After rinsing with distilled water, the bananas were sliced into 2 mm sections and treated with 0.5% (*w*/*v*) citric acid solution to decrease enzymatic browning before draining. The slices were initially frozen at −80 °C for 4 h and subsequently freeze-dried using a Zirbus VaCo 5 System (Zirbus, Bad Grund, Germany) at −50 °C and 0.5 hPa for 72 h. The dried material was milled into powder with a Breville Smart Grinder TM Pro (model BCG820BSSXL, Melbourne, VIC, Australia), producing flour with an average particle size of 200 µm. The flour was packaged in sealed plastic containers and stored at 4 °C until analysis.

### 3.2. Preparation of Banana-Enriched Bread

Using the procedure utilized in our previous work [57], bread was made in the Food Processing Laboratory, Faculty of Science, School of Agriculture, Food and Ecosystem Sciences, University of Melbourne, Australia following the Basic Straight-Dough Bread-Baking Method (AACC Method 10-09.01) [89]. The formulation consisted of wheat flour with or without substitution, 63–76% water, 2% salt, 2% yeast, 4% sugar, and 3% fat, with proportions calculated relative to flour weight. All ingredients were mixed in a stand mixer (Kenwood Titanium Chef Baker XL Stand Mixer White KVL65001WH, Havant, UK) at low speed for 3 min, followed by 10 min at medium speed, and finally an additional 10 min at high speed until the dough was smooth and elastic. The dough was then subjected to bulk fermentation for 60 min at 27–30 °C, during which it doubled in size. After fermentation, it was degassed, shaped, and proofed for another 60 min at 30–35 °C.

Subsequently, the bread was baked at 170 °C for 60 min. After baking, loaves were cooled at room temperature (21–25 °C) for 1 h before slicing or packaging. Whole banana flours were incorporated at substitution levels of 5%, 10%, and 15% (*w*/*w*), whereas a control bread was prepared with 100% wheat flour. Notably, GBF was introduced during the dough-mixing stage to ensure uniform incorporation. Finally, both control and banana-enriched breads were freeze-dried (Zirbus VaCo 5 System, Bad Grund, Germany), ground into fine powder using a coffee grinder (Breville Smart Grinder Pro, model BCG820BSSXL, Melbourne, VIC, Australia), and stored at −20 °C until further analysis.

### 3.3. Phenolic Compounds’ Extraction

The extraction of free phenolic compounds from wheat–banana bread samples was carried out following the method of Peng et al. [90] with minor modifications. In brief, two grams of powdered sample were mixed with 20 mL of 80% ethanol, prepared by diluting absolute ethanol with Milli-Q water, and each extraction was performed in triplicate. The mixtures were placed in an incubator shaker (ZWYR-240 incubator shaker, Labwit, Ashwood, VIC, Australia) and agitated at 150 rpm for 16 h at 20 °C to promote the release of phenolic compounds. After incubation, the samples were centrifuged at 8000 rpm for 15 min (ROTINA380R, Hettich Refrigerated Centrifuge, Tuttlingen, Baden-Württemberg, Germany). The resulting supernatant was carefully collected, passed through a 0.45 µm syringe filter to remove particulates, and subsequently stored at −20 °C until further analysis [28].

### 3.4. In Vitro Gastrointestinal Digestion

The GI digestion of GBF-enriched wheat bread was conducted with slight modifications to the protocols of Gu et al. [84]. Simulated oral (SOF), gastric (SGF), and intestinal (SIF) fluids were prepared following the harmonized INFOGEST 2.0 static in vitro guidelines. Bread powder was blended with distilled water at a 2:1 (*w*/*v*) ratio, from which a 5 mL aliquot was collected as the undigested control. Subsequently, the oral phase was initiated by adding SOF (1:1, *v*/*v*), adjusting the pH to 7.0, and incorporating salivary amylase (75 U/mL). The mixture was incubated at 37 °C while stirring for 2 min, after which a 5 mL aliquot was taken as the oral digest. Next, the gastric phase was simulated by combining the oral bolus with SGF (1:1, *v*/*v*) containing porcine pepsin (2000 U/mL). The pH was then reduced to 3.0 using HCl, and the mixture incubated for 2 h at 37 °C. At the end of this stage, a 5 mL aliquot was collected, and the pH was subsequently readjusted to 7.0 to terminate gastric digestion. Finally, the intestinal phase was initiated by adding SIF (1:1, *v*/*v*) supplemented with trypsin (100 U/mg) and bile salts (10 mM). The mixture was incubated for 2 h under the same conditions. Thereafter, aliquots from each digestion phase were rapidly frozen in liquid nitrogen to halt enzymatic reactions and stored for later analysis [28].

### 3.5. In Vitro Colonic Fermentation

Following the procedures described by Gu et al. [84] and Bashmil et al. [28], the non-absorbed fraction remaining after GI digestion was subjected to colonic fermentation under conditions simulating the human gut microbiota. Pig feces were employed as a substitute for human feces. Samples were collected from ten male and female finisher white pigs (approximately 50 kg live weight) housed at Diamond Valley Pork (Thomas Road, Laverton North, Victoria, Australia) and maintained on a standard diet for two weeks. Fresh fecal samples were pooled in an anaerobic chamber and homogenized to prepare a suspension (20% *w*/*w*), consisting of 20 g feces and 80 g phosphate buffer (pH 7.0). Homogenization was carried out for 5 min using a stomacher blender (MiniMix^®^ Lab Blender, Thomas Scientific, Swedesboro, NJ, USA). The blended fecal slurry was passed through a muslin layer to remove coarse material before use. For fermentation, 5 mL of intestinal digesta was mixed with 5 mL of fecal inoculum and basal media and placed into six sets of test tubes. These were flushed with nitrogen to maintain anaerobic conditions and incubated in the dark at 37 °C, with agitation at 120 rpm, for 0, 3, 6, 12, 24, and 48 h. After incubation, supernatants were obtained by centrifugation (10,000× *g*, 10 min, 4 °C) and stored for subsequent measurement of SCFAs production and evaluation of phytochemical bioactivity.

### 3.6. Estimation of Phenolic Content and Antioxidant Capacity

#### 3.6.1. Total Phenolic Content (TPC)

The TPC of GBF-enriched bread samples was determined following the procedure of Mussatto et al. [91] with minor modifications. Briefly, either sample extract or gallic acid standard solution (0–200 g/mL) was sequentially combined with Folin–Ciocalteu’s reagent and distilled water (1:1:8, *v*/*v*/*v*) in a 96-well plate. The mixture was incubated for 5 min at 25 °C in the absence of light. Then, 10% (*w*/*w*) sodium carbonate was added in equal volume to the sample solution, followed by a further 1 h incubation under the same conditions. Absorbance was recorded at 765 nm using a UV–VIS spectrophotometer (Thermo Fisher Scientific, Waltham, MA, USA). Results were expressed as mean mg gallic acid equivalents (GAE) per gram dry weight (mg GAE/g ± standard deviation (SD)) based on three independent assays [28].

#### 3.6.2. Total Flavonoid Content (TFC)

The TFC of GBF bread samples was estimated following the procedure of Suleria et al. [92] with slight modifications. Briefly, aliquots of sample extract or quercetin standard (0–50 g/mL), 2% aluminum chloride, and 50 g/L sodium acetate (1:1:1.5, *v*/*v*/*v*) were sequentially dispensed into a 96-well microplate. The mixtures were incubated for 2.5 h at 25 °C in the dark, after which absorbance was measured at 440 nm. Results from three independent assays were expressed as mean milligrams of quercetin equivalents per gram of dry weight (mg QE/g) ± standard deviation (SD) [28].

#### 3.6.3. Total Tannins Content (TTC)

The TTC of GBF bread samples was assessed using the method described by Suleria et al. [92]. In brief, sample extracts or catechin standards (0–1 mg/mL), vanillin solution, and 32% sulfuric acid (1:6:1, *v*/*v*/*v*) were sequentially added to a 96-well plate. The mixtures were incubated for 15 min at 25 °C in darkness, and absorbance was recorded at 500 nm. The results from three replicates were displayed as mean milligrams of catechin equivalents per gram of dry weight (mg CE/g) ± standard deviation (SD) [28].

#### 3.6.4. 2,2-Diphenyl-2-picryl-hydrazyl (DPPH)

The free radical scavenging capacity of GBF bread extracts was determined according to the method of Suleria et al. [92]. DPPH solution was prepared by mixing dye and methanol (1:25, *w*/*w*) to obtain a 0.1 mM radical solution. For each reaction, 40 µL of extract or Trolox standard (0–200 µg/mL) and 260 µL of the DPPH solution were pipetted into a 96-well plate and incubated in the dark at 25 °C for 30 min. Absorbance was then recorded at 517 nm. The findings of three independent assays were expressed as mean milligrams of Trolox equivalents (mg TE/g dry weight) ± standard deviation (SD) [28].

#### 3.6.5. Ferric Reducing Antioxidant Power (FRAP)

The FRAP of GBF bread samples was measured following the method of Suleria et al. [92]. The FRAP dye solution was freshly prepared, in the dark, by mixing 300 mM sodium acetate, 10 mM TPTZ solution, and 20 mM Fe [III] solution at a ratio of 10:1:1 (*v*/*v*/*v*). Briefly, 20 μL of sample extract or Trolox standard (0–200 g/mL) was combined with 280 μL of dye solution in a 96-well plate and incubated at 37 °C for 10 min. Absorbance was read at 593 nm, and outcomes were reported as mean milligrams of Trolox equivalents (mg TE/g dry weight) ± SD from triplicate assays [28].

### 3.7. Phenolic Compounds Quantification via High-Performance Liquid Chromatography Photodiode Array (HPLC-PDA)

The quantification of targeted phenolic compounds in banana bread flour was conducted using Agilent 1200 series HPLC (Agilent Technologies, Santa Clara, CA, USA) equipped with a photodiode array (PDA) reader, following the protocol described by Suleria et al. [92] with minor modifications [28]. Prior to analysis, sample extracts were filtered through a 0.45 μm syringe filter (PVDF, Millipore, MA, USA). Chromatographic separation was achieved using a Synergi Hydro-RP column (250 mm length × 4.6 mminternal diameter (i.d.)) with a 4 µm particle size (Phenomenex, Lane Cove, NSW, Australia), protected by a Phenomenex C18 ODS guard column (4.0 mm × 2.0 mm i.d.). Each sample or standard (20 μL) was injected for analysis. The mobile phases were consisted of water: acetic acid (98:2, *v*/*v*) (phase A) and acetonitrile–water–acetic acid (55:43:2, *v*/*v*/*v*) (phase B). The range of gradient profile was as follows: 10–25% B (0–20 min), 25–35% B (20–30 min), 35–40% B (30–40 min), 40–55% B (40–70 min), 55–80% B (70–75 min), 80–90% B (75–77 min), 90–100% B (77–79 min), 100–10% B (79–82 min), isocratic 10% B (82–85 min). The flow rate was maintained at 0.8 mL/min under ambient column temperature. PDA detection was performed at the wavelengths 280, 320, and 370 nm simultaneously. Empower Software (2010 version) was employed for device management, data acquisition, and chromatographic evaluation.

### 3.8. Gastrointestinal Digestion and Colonic Fermentation Parameters

#### Bio-Accessibility of Phenolic Compounds

The bio-accessibility of each phenolic compound was calculated as the quantity of compound released from samples at each digestion phase and accumulated in the residual fraction throughout the GI in vitro digestion process and colonic fermentation. Bio-accessibility (%) was calculated using the following Formulas (1)–(4):

Oral Bio-accessibility (%) = (Oral fraction/Total phenolic amount) × 100(1)

Gastric Bio-accessibility (%) = (Gastric fraction/Total phenolic amount) × 100(2)

Intestinal Bio-accessibility (%) = (Intestinal fraction/Total phenolic amount) × 100(3)

Colonic Bio-accessibility (%) = (Colonic fraction/Total phenolic amount) × 100(4)

### 3.9. Evaluation of Short Chain Fatty Acids (SCFAs)

The determination of SCFAs was conducted with slight modifications to the previously reported protocol of Gu et al. [84]. Briefly, 1 g of material from each stage of gut fermentation was mixed with 5 mL of water and vortexed for 3 min. The mixtures were then acidified with 5 mol/L HCl to reach a pH of approximately 2.0, followed by centrifugation at 10,000 rpm and 10 °C for 10 min. From each supernatant, 1 mL was mixed with 4 mL of diluted acid solution containing 1% formic acid and 1% orthophosphoric acid and blended thoroughly. The 4-methyl-valeric acid solution with concentration of 1.59 mmol/L was included as an internal standard. Calibration standard curves were prepared using acetic, propionic, butyric, isobutyric, and valeric acids. All standards and chemicals were stored at 4 °C until analysis. For SCFAs analysis, colon digesta samples were analyzed by gas chromatography (7890B Agilent, Santa Clara, CA, USA) equipped with a flame ionization detector (GC-FID), an autosampler (Gilson GX-271, Gilson Inc., Middleton, WI, USA) and autoinjector. The SCFAs were separated on a capillary column (SGE BP21, 12 nm × 0.53 nm i.d.) with 0.5 µm film thickness, SGE International, Ringwood, VIC, Australia, P/N 054473) with retention gap kit (including a 2 mm × 0.53 mm i.d. guard column, P/N SGE RGK2). The injection volume was 1 μL. Helium was used as carrier gas at a flow rate of 14.4 mL/min, while the composition gas consisted of nitrogen, hydrogen, and air with flow rates of 20, 30, and 300 mL/min, respectively. The oven was initially set at 100 °C for 30 s, then increased to 180 °C for 1 min at 6 °C/min, then raised to 200 °C for 10 min at 20 °C/min. Detector and injector port temperatures were maintained at 240 °C and 200 °C, respectively. SCFA concentrations were expressed in mmol/L for statistical analysis [28].

### 3.10. Statistics Analysis

The results of this research were expressed as the means ± standard deviation (SD) based on three independent replicates. Statistical significance was assessed using two-way analysis of variance (ANOVA) followed by Tukey’s post hoc test to evaluate differences among GBF-enriched breads across substitution levels and digestion phases with respect to phenolic content and antioxidant activity (*p* ≤ 0.05). Data analyses and graphical outputs were carried out using Minitab 19 (Minitab^®^ for Windows, Release 19; Minitab Inc., Chicago, IL, USA) and GraphPad Prism 10 (Version 10.0.2; GraphPad Software Inc., San Diego, CA, USA). To ensure reliability, all reported values were corrected by subtracting corresponding blank or control measurements.

## 4. Conclusions

In conclusion, the in vitro GI digestion and colonic fermentation facilitated by digestive enzymes and gut microbiota can effectively improve the bio-accessibility of phenolic compounds and enhance their antioxidant characteristics in GBF-enriched bread. Generally, the bio-accessibility of various phenolic compounds can vary based on variations in banana cultivars and substitution levels, with intestinal digestion resulting in the highest bio-accessibility. Even after 48 h of colonic fermentation, phenolic bio-accessibility remains significantly measurable. The breakdown of complex phenolic compounds during digestion may retain or even increase the antioxidant capacity of banana bread. Further, the high polyphenol content of the GBF bread contributes to its ability to produce significant concentrations of SCFAs during 24 h fecal fermentation. These findings highlight the promising role of banana-enriched bread as a functional food, offering both prebiotic effects and antioxidant benefits. However, further studies are needed to thoroughly understand how the metabolic transformations of phenolic compounds, influenced by insoluble DF, starch fractions, and gluten, may enhance polyphenol bioactivity and retention in functional bakery products.

## Figures and Tables

**Table 1:** Quantification of phenolic compounds in wheat-banana enriched bread by HPLC-PDA analysis.

Bread Samples	Substitution %	Phenolic Compounds (µg/g Dry Weight)															
		Gallic Acid	Protocatechuic Acid	Caftaric Acid	*p*-hydroxybenzoic Acid	Catechin	Chlorogenic Acid	Caffeic Acid	Syringic Acid	Epicatechin	Coumaric Acid	Polydatin	Diosmin	Resveratrol	Quercetin	Kaempferol	Total Phenolics
Control	0	109 ± 1.1 ^f^	332 ± 0.4 ^i^	56.9 ± 1.0 ^h^	77.7 ± 1.2 ^h^	92.7 ± 2.0 ^j^	11.1 ± 1.1 ^h^	5.3 ± 0.3 ^g^	23.1 ± 0.2 ^i^	81.0 ± 0.5 ^j^	6.1 ± 0.1 ^e^	0.8 ± 0.1 ^e^	2.3 ± 0.3 ^g^	3.4 ± 0.7 ^g^	17.5 ± 0.6 ^g^	10.5 ± 0.6	829 ± 2.5 ^j^
Cavendish	5	123 ± 0.8 ^d^	453 ± 0.5 ^e^	123 ± 1.5 ^e^	94.4 ± 1.2 ^c^	150 ± 0.8 ^g^	111 ± 1.1 ^e^	6.7 ± 0.8 ^g^	33.7 ± 0.3 ^g^	160 ± 0.4 ^e^	18.0 ± 0.6 ^cd^	2.3 ± 0.3 ^b-d^	7.0 ± 0.1 ^de^	12.2 ± 0.4 ^cd^	21.0 ± 1.0 ^ef^	11.9 ± 0.2	1327 ± 5.3 ^g^
Ladyfinger	5	98.3 ± 2.1 ^g^	414 ± 0.8 ^g^	106 ± 0.3 ^g^	97.1 ± 1.5 ^i^	122 ± 1.7 ^i^	76.0 ± 0.3 ^g^	13.4 ± 0.3 ^f^	29.1 ± 0.4 ^h^	106 ± 0.4 ^i^	12.0 ± 0.1 ^cd^	1.2 ± 0.4 ^de^	4.4 ± 0.2 ^f^	4.7 ± 1.4 ^g^	19.8 ± 0.9 ^fg^	12.6 ± 0.1	1115 ± 3.8 ^i^
Ducasse	5	93.5 ± 1.0 ^h^	473 ± 0.9 ^d^	183 ± 1.2 ^c^	467 ± 1.8 ^j^	266 ± 0.9 ^d^	128 ± 1.7 ^d^	25.7 ± 0.9 ^c^	39.8 ± 0.3 ^e^	153 ± 0.5 ^f^	17.4 ± 0.7 ^cd^	3.5 ± 0.1 ^b^	5.6 ± 0.2 ^ef^	12.7 ± 0.9 ^c^	22.3 ± 0.3 ^ef^	12.3 ± 0.4	1902 ± 1.3 ^c^
Cavendish	10	120 ± 0.3 ^d^	450 ± 0.9 ^f^	142 ± 1.0 ^d^	215 ± 1.1 ^b^	174 ± 0.9 ^f^	130 ± 0.6 ^d^	20.1 ± 0.6 ^de^	43.6 ± 0.4 ^d^	171 ± 1.0 ^d^	21.0 ± 1.0 ^cd^	2.9 ± 0.1 ^bc^	7.8 ± 0.4 ^cd^	12.4 ± 0.2 ^c^	23.2 ± 0.7 ^de^	18.3 ± 1.0	1551 ± 6.9 ^e^
Ladyfinger	10	116 ± 0.2 ^e^	498 ± 0.4 ^c^	194 ± 1.1 ^b^	118 ± 1.2 ^f^	190 ± 0.6 ^e^	177 ± 0.4 ^c^	27.9 ± 0.1 ^b^	110 ± 1.1 ^b^	143 ± 0.6 ^g^	20.2 ± 0.4 ^c^	1.5 ± 0.4 ^ce^	8.8 ± 1.0 ^c^	7.0 ± 0.2 ^f^	23.6 ± 1.0 ^de^	24.6 ± 1.5	1660 ± 3.6 ^d^
Ducasse	10	83.0 ± 0.2 ^i^	392 ± 1.1 ^h^	112 ± 1.7 ^f^	143 ± 1.0 ^g^	135 ± 1.0 ^h^	104 ± 2.2 ^f^	18.5 ± 0.7 ^e^	36.6 ± 0.2 ^f^	123 ± 0.9 ^h^	14.5 ± 0.9 ^d^	2.8 ± 0.4 ^bc^	5.3 ± 1.2 ^ef^	9.4 ± 1.2 ^e^	25.9 ± 0.1 ^d^	13.9 ± 1.3	1219 ± 2.2 ^h^
Cavendish	15	165 ± 0.9 ^c^	2462 ± 0.8 ^a^	187 ± 2.0 ^c^	1536 ± 0.3 ^a^	2148 ± 2.0 ^a^	252 ± 0.8 ^b^	39.2 ± 0.1 ^a^	62.5 ± 0.5 ^c^	387 ± 0.9 ^a^	246 ± 0.6 ^a^	6.6 ± 0.3 ^a^	15.5 ± 0.4 ^a^	32.6 ± 1.0 ^a^	71.0 ± 0.2 ^a^	91.6 ± 1.0	7699 ± 7.3 ^a^
Ladyfinger	15	456 ± 1.0 ^a^	1160 ± 0.8 ^b^	389 ± 1.2 ^a^	233 ± 2.1 ^d^	379 ± 0.1 ^b^	645 ± 2.1 ^a^	38.8 ± 0.3 ^a^	119 ± 0.2 ^a^	209 ± 1.1 ^c^	26.2 ± 1.2 ^b^	3.2 ± 0.2 ^b^	13.5 ± 0.8 ^b^	18.7 ± 0.6 ^b^	41.4 ± 0.6 ^b^	60.3 ± 1.0	3460 ± 0.6 ^b^
Ducasse	15	239 ± 0.5 ^b^	225 ± 2.4 ^j^	109 ± 2.2 ^fg^	122 ± 2.4 ^e^	327 ± 1.1 ^c^	112 ± 0.2 ^e^	20.8 ± 1.6 ^d^	32.4 ± 0.4 ^g^	265 ± 2.2 ^b^	15.1 ± 1.1 ^cd^	2.6 ± 1.2 ^b–d^	5.1 ± 0.1 ^f^	10.2 ± 0.7 ^de^	29.5 ± 1.2 ^c^	17.6 ± 2.0	1528 ± 7.7 ^f^

Values are expressed as mean ± standard deviation (µg/g dry weight). Values in the same column with various superscript letters (^a–j^) are significantly different from each other (*p* < 0.05).

**Table 2:** Evaluation of phenolic compounds bio-accessibility in 10% green banana-enriched bread following in vitro digestion and colonic fermentation.

No	Compound	Oral BIA (%)				Gastric BIA (%)				Intestinal BIA (%)				Colonic BIA (%)			
		0%	10% C	10% L	10% D	0%	10% C	10% L	10% D	0%	10% C	10% L	10% D	0%	10% C	10% L	10% D
1	Gallic acid	92.8	101	95.7	84.2	103	111	104	97.4	101	98.3	91.4	95.2	19.0	87.4	86.2	78.5
2	Protocatechuic acid	87.8	93.5	90.4	77.0	58.2	95.8	97.2	87.2	95.0	98.0	98.4	91.0	29.1	46.9	76.5	56.4
3	Caftaric acid	19.2	11.3	26.8	15.2	49.2	63.7	58.4	74.0	61.4	101	86.2	96.3	17.0	56.9	49.5	62.3
4	*p*-hydroxybenzoic acid	93.8	100	93.6	98.4	68.1	105	90.1	97.0	93.3	99.9	93.5	90.1	25.8	37.5	42.5	31.8
5	Catechin	28.0	34.6	23.8	31.8	46.1	57.9	48.2	53.1	96.4	92.3	64.1	75.3	3.91	16.2	34.4	26.0
6	Chlorogenic acid	13.5	32.7	35.3	40.0	20.7	69.8	71.4	59.3	49.6	88.8	78.7	72.7	32.5	12.0	27.4	15.0
7	Caffeic acid	30.5	25.5	29.2	22.4	39.9	77.2	64.9	54.7	72.1	90.2	71.4	81.7	21.8	20.7	36.5	49.5
8	Syringic acid	90.5	82.4	75.2	59.9	86.2	92.0	81.5	87.2	99.2	96.2	88.8	92.7	24.1	27.5	33.5	24.5
9	Epicatechin	11.6	15.1	12.4	19.0	16.4	59.2	47.0	32.9	51.0	80.0	86.8	90.7	4.34	8.51	14.3	10.2
10	Coumaric acid	81.0	71.2	49.2	61.7	97.4	82.4	54.2	61.7	81.0	85.5	74.0	75.5	32.2	33.2	40.4	27.3
11	polydatin	0.00	13.7	8.44	17.7	24.6	32.6	26.5	42.7	32.0	68.9	59.8	53.5	7.43	17.2	21.3	14.3
12	Diosmin	7.17	27.5	24.5	19.7	9.36	35.9	17.6	38.6	54.1	76.2	67.6	72.6	0.00	3.26	6.31	1.07
13	Resveratrol	5.30	16.0	9.74	21.1	17.1	40.7	28.3	21.1	58.2	56.3	42.6	44.5	2.67	16.1	11.4	21.2
14	Quercetin	0.00	19.9	15.3	7.34	26.3	38.7	40.7	52.5	37.7	58.7	66.1	60.3	0.00	37.3	28.2	45.0
15	Kaempferol	2.36	28.2	16.9	22.7	30.0	58.0	37.2	51.5	39.5	71.9	81.9	65.8	0.00	50.1	39.0	66.0
	Total phenolic compounds	64.7	62.7	57.9	55.9	58.0	82.2	75.4	73.4	86.2	93.4	86.6	86.6	20.1	37.3	50.3	41.9

C: Cavendish; L: Ladyfinger; D: Ducasse.

## Data Availability

The data presented in this study are available in this manuscript.
